# Effects of a polyphenol-rich extract blend, probiotics, and hydrolyzed fiber on quality of life and gut health markers in patients with irritable bowel syndrome—A randomized, double-blind, placebo-controlled trial

**DOI:** 10.3389/fnut.2025.1603011

**Published:** 2025-07-07

**Authors:** Adrianna Wierzbicka, Barbara Khaidakov, Oliwia Zakerska-Banaszak, Paulina Andrzejewska, Alina Baturo, Paulina Kowalczyk, Krzysztof Lemke, Agnieszka Dobrowolska, Marzena Skrzypczak-Zielinska, Dorota Mankowska-Wierzbicka

**Affiliations:** ^1^Department of Gastroenterology, Dietetics and Internal Diseases, Poznan University of Medical Sciences, Poznan, Poland; ^2^Department of Internal Medicine, Cleveland Clinic Florida, Weston, FL, United States; ^3^R&D Department, AronPharma Ltd., Gdańsk, Poland; ^4^Institute of Human Genetics, Polish Academy of Sciences, Poznań, Poland; ^5^3P-Medicine Laboratory, Medical University of Gdańsk, Gdańsk, Poland

**Keywords:** irritable bowel syndrome, dietary intervention, probiotics, fiber, polyphenols, quality of life, SCFAs, FODMAP

## Abstract

**Introduction:**

Irritable bowel syndrome (IBS) is the most prevalent functional bowel disorder impacting around 5%−10% of the general population worldwide. The pathogenesis remains unclear, however alterations in gut-brain axis play a critical role. We aimed to investigate the therapeutic potential of a novel synbiotic formulation comprising of partially hydrolyzed guar gum (PHGG), specific probiotic strains (*Bifidobacterium* and *Saccharomyces boulardii*), and double-standardized, polyphenol-rich blend of extracts from *Aronia melanocarpa* and *Sambucus nigra* in patients with IBS.

**Methods:**

A total of 47 patients with IBS were randomly assigned to three groups and followed over a 2-month study period. Group I (*n* = 14) received placebo capsules, Group II (*n* = 14) took one placebo capsule along with a probiotic formulation and PHGG, Group III (*n* = 19) received probiotic formulation, PHGG and polyphenol-rich fruit extracts blend. The IBS-quality of life (QoL) questionnaire was completed by all participants at baseline and after 2 months. Serum levels of IL-6, IL-8, TNF-α, I-FABP-2, GM-CSF and stool concentrations of short-chain fatty acids (SCFAs) and zonulin were evaluated before and after intervention.

**Results and conclusions:**

This study demonstrated a significant improvement in QoL in individuals receiving the complete formulation combination (Group III). The largest decrease in score was observed in dysphoria, with median differences of −5 in Group III (*p* = 0.0021), −3 in Group II (*p* = 0.0155), and −1 in the control Group I (*p* = 0.0338). Significant correlations were found in Groups II and III between supplementation and serum concentrations of IL-8, TNF, and GM-CSF (*p* < 0.05). A significantly higher concentration of all SCFAs was seen after intervention in Group III compared to control Group I.

## 1 Introduction

The intestine serves not only as a digestive absorptive organ, encompasses neural tissue corresponding to that of the entire spinal cord, but is also considered as one of the largest immune organs in the body. Irritable bowel syndrome (IBS) is a common functional disorder characterized by persistent or intermittent abdominal pain, bloating pertaining to relief or exacerbation by defecation, or alteration in bowel habits. IBS is the most prevalent functional bowel disorder impacting around 5%−10% of the general population worldwide (1). Based on the Rome IV criteria, we classify four subtypes of IBS: diarrhea-predominant (IBS-D), constipation-predominant (IBS-C), mixed (IBS-M), and unsubtyped (IBS-U) (2). The pathogenesis entails gut-brain interactions, enteric microbiota alterations, visceral hypersensitivity, genetic factors (mutations of *SCN5A* gene), disordered bile salt metabolism, low-grade mucosal inflammation, and dysregulated intestinal permeability (3). Given the variability of IBS symptoms, pharmacological therapy is recommended to target specific symptoms like abdominal pain, diarrhea or constipation. Dietary modifications, such as low FODMAP (low in fermentable oligosaccharides, disaccharides, monosaccharides, and polyols) diet, are frequently advocated as first line treatment option. Examples of low FODMAP foods include carrots, cucumbers, zucchini, white rice, oats, unripe bananas, eggs, firm tofu, and lactose-free dairy products. Conversely, high FODMAP foods commonly avoided include onions, garlic, apples, pears, wheat-based bread, legumes, and milk. Studies report 52%−86% of patients experiencing significant symptom improvement with FODMAP restriction (4). The diet has demonstrated effectiveness in reducing abdominal pain, bloating, and flatulence (5, 6). However, long-term adherence requires careful management to avoid nutritional deficits and adverse effects on gut microbiome (7). Implementation should involve a structured approach with restriction, reintroduction, and personalization phases, ideally supervised by a dietician (7). Although the low FODMAP diet appears promising, further research is necessary to clarify its long-term impact and determine the most effective ways to implement it (5, 6, 8, 9).

There is growing evidence that functional dietary supplements such as polyphenols, fiber, and probiotics have a beneficial effect on symptom management in IBS patients. Polyphenols are plant-based compounds, commonly found in diverse selection of fruits and vegetables. They exert antispasmodic, anti-inflammatory, antioxidant properties, modulate cytokine production, and inflammatory pathways (6, 10). Partially hydrolyzed guar gum (PHGG) is a water-soluble fiber, which not only plays a crucial role in bowel movement regulation, contributes to short-chain fatty acids (SCFAs) production, but is also effective in postprandial glucose management (11). Probiotics are live non-pathogenic microorganisms which exert their beneficial effects through various mechanisms, including reducing colonization and invasion by pathogens, lowering intestinal pH, and modifying host immune response (12). They have been proven to improve stool consistency, reduce the colonic transit time, lower the average number of daily bowel movement, improving the overall quality of life.

Over the past four decades, there has been a growing recognition of the importance of patient-centered outcome data in evaluating the impact of chronic and debilitating diseases, including IBS. The IBS-quality of life (IBS-QoL) questionnaire is a specific, validated tool assessing eight major domains found to be imperative to individuals with IBS, including dysphoria, interference with activity, body image, health worry, food avoidance, relationships, social reaction, and sexual relations (13). It has been used worldwide in both the clinical and research practice to evaluate disease burden and treatment outcomes. Interestingly, data has reported markedly reduced QoL in individuals with IBS compared to those with diabetes mellitus, gastroesophageal reflux or dialysis depended end-stage renal disease (14).

Thus, prompt and careful identification of the variables that describe reduction in QoL are required to be address and successfully treated.

In this study, we aim to investigate the therapeutic potential of a novel synbiotic formulation comprising of partially hydrolyzed guar gum (PHGG), selected probiotic strains (*Bifidobacterium* and *Saccharomyces boulardii*), and polyphenol-rich fruit extracts blend Fenactive^®^ (*Aronia melanocarpa* and *Sambucus nigra*) in individuals with IBS. We strongly believe that the synergistic combination of these ingredients will contribute to significant improvement in patient's QoL and relevant biomarkers. Moreover, these findings will shed light into dietary strategies, providing potential avenues for future clinical implication.

## 2 Materials and methods

### 2.1 Study design

This was a randomized, double-blind, placebo-controlled trial conducted in a single research center in Poland. The first patient was enrolled on 26. 04. 2023, and the last patient on 23. 10. 2023. The trial was registered on clinicaltrials.gov with the identifier NCT05990764 on August 07, 2023. Full details can be accessed at clinicaltrials.gov/study/NCT05990764. A total of 47 individuals with IBS were recruited and randomly assigned to three groups: Group I (*n* = 14), Group II (*n* = 14), and Group III (*n* = 19). Participants were screened based on the established diagnostic requirements for IBS. Inclusion criteria were medically diagnosed IBS (subjects with recognizable symptoms of IBS, including recurrent abdominal pain occurring on average at least 1 day per week over the past 3 months), symptoms fulfilling at least 2 of the 3 criteria: (a) pain related to defecation; (b) pain associated with change in frequency of bowel movements; (c) pain associated with change in stool consistency. The patients were aged between 18 and 65 years, willing and able to provide informed consent to participate in the study. Subjects expressed their eagerness to comply with the study procedures and follow the prescribed intervention regimen. Individuals, who were currently using supplements containing plant extracts, polyphenols, anthocyanins, fiber, probiotics, or prebiotics were excluded from the study. Exclusion criteria included participation in another clinical trial, inability to swallow oral medication/placebo, presence of serious medical conditions such as cancer, autoimmune disorders, severe liver dysfunction, tuberculosis, leukemia, multiple sclerosis, AIDS, rheumatoid arthritis, organ transplant, or other gastrointestinal diseases that could impact study outcomes, as well as pregnancy or pregnancy planning. Moreover, individuals with any clinically relevant medical history or current condition that could interfere with data interpretation or pose safety concerns, as determined by the principal investigator and/or co-investigator, were excluded from the study.

Our study was carried out in compliance with ethical protocols and received approval from the Bioethics Committee of the Poznan University of Medical Sciences (decision no. 110/23). Informed consent was obtained from all participants prior to their enrollment in the study.

### 2.2 Intervention

The patients enrolled in the study were in the post-personalization phase of the low FODMAP diet (fermentable oligo-, di-, and monosaccharides and polyols), having previously completed both the restriction and reintroduction phases. The supplementation administered during the study was intended to support the gut microbiota following diet personalization. Prior to enrollment, patients exhibited varying degrees of tolerance to fructans (e.g., those found in wheat) and the disaccharide lactose. At the time of inclusion, all participants were regularly consuming all categories of FODMAPs, including polyols, fructose, fructans, galactans, and lactose. Participants in Group I (Placebo) received two placebo capsules and one sachet containing an inactive substance (maltodextrin). Participants in Group II obtained one placebo capsule along with a probiotic formulation containing: *Bifidobacterium animalis* subsp. *lactis* BLC1 (5 × 10^9^ CFU) obtained from SACCO System, Codargo, Italy, *Saccharomyces boulardii* SP9 (250 mg) obtained from SACCO System, Codargo, Italy, *Bifidobacterium* lactis UABla-12TM (1 × 10^10^ CFU) obtained from CHR HANSEN, Hoersholm, Denmark, and *B. animalis* subsp. *lactis* BS01 (LMG P-21384; 5 × 10^9^ CFU) obtained from Probiotical, Novara, Italy. Additionally, participants also received 5 g of partially hydrolyzed guar gum (Sunfiber^®^ A, ProAgro GmbH, Vienna, Austria). Participants in Group III were given 200 mg of Fenactive^®^ (an original blend of double-standardized extracts of black chokeberry (*A. melanocarpa*) and elderberry (*S. nigra)* fruit extracts, proprietary composition—Fenactive^®^, provided by Greenvit Botanical Extracts Manufacturer, Zambrów, Poland), standardized to total polyphenols (25% = 50 mg), and total anthocyanins (15% = 30 mg). They also received PHGG (5 g) and the same probiotic formulation as Group II. Each enrolled participant was provided with a daily intervention for a total duration of 2 months.

### 2.3 Quality of life assessment

A total of 45 participants (14 from Group I, 14 from Group II, and 17 from Group III) completed the IBS-QoL questionnaire at baseline (T0) and after 2 months (T1). This is a detailed survey, consisting of 34 items, assessing various aspects of life affected by IBS symptoms on a scale of 1–5. For analysis, responses from the IBS-QoL questionnaire were grouped into eight domains: dysphoria, activity interference, body image, health worry, food avoidance, social reaction, sexual concerns, and relationships (13). Each domain consists of specific questionnaire items, with full item-domain mappings provided in Supplementary Table 1.

### 2.4 Blood sampling and analysis

**Figure 1:**
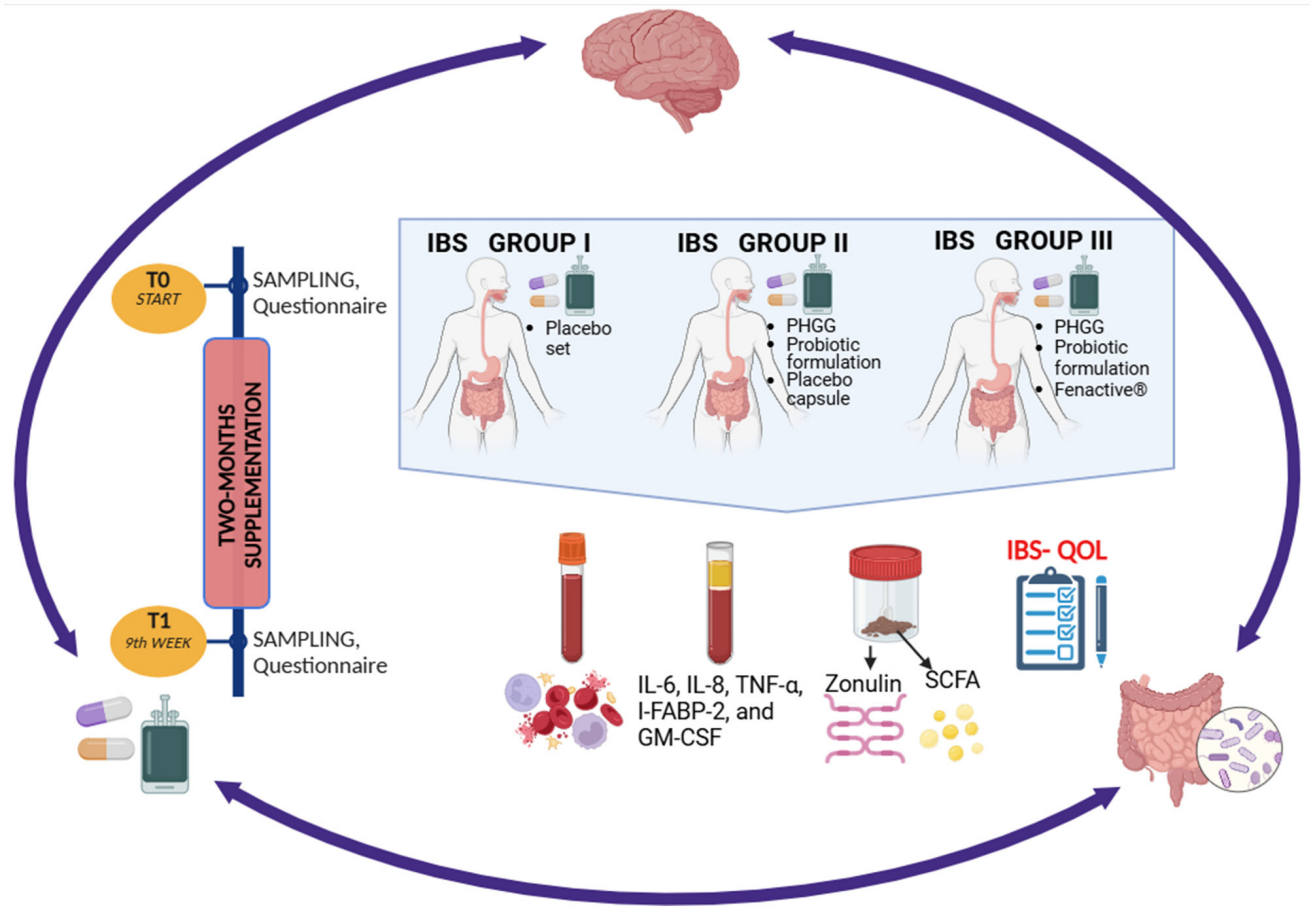
Schematic overview of the study design. Created with BioRender.com. Skrzypczak-Zielinska, M. (2025) https://BioRender.com/d13p202.

Blood samples were collected from all participants at baseline (T0) and after 2 months (T1). The complete blood count analysis (ICD-9: C55) was conducted by Diagnostyka S. A. diagnostic laboratory (Poznan, Poland) upon request. Levels of inflammatory markers, including IL-6, IL-8, TNF-α, I-FABP-2, and GM-CSF, were measured in the serum using enzyme-linked immunosorbent assay (ELISA) kits according to the manufacturer's protocols. The absorbance was measured at 450 nm wavelength (except for I-FABP-2, which was measured at 405 nm) utilizing an EL-808 scanner spectrometer (BioTek Instruments Inc., USA). Reagent kits included Human IL-6 HS ELISA Kit Diaclone, cat. no. 950.035.192, test sensitivity 0.81 pg/ml, detection range 1.56–50 pg/ml; Human IL-8 ELISA Kit Diaclone, cat. no. 950.050.192, test sensitivity 12.3 pg/ml, detection range 31.25–1000 pg/ml; Human TNF alpha ELISA Kit Diaclone, cat. no. 950.090.192, test sensitivity 8 pg/ml, detection range 25–800 pg/ml; Human GM-CSF ELISA Kit Diaclone, cat. no. 873.040.192, test sensitivity 4.8 pg/ml, detection range 15.6–500 pg/ml; Intestinal FABP (I-FABP-2) Human ELISA Biovendor, cat. no. RD191246200R, test sensitivity 3.5 pg/ml, 20–1280 pg/ml. There were two missing blood samples during T1 (one from Group I and one from Group II), which were accounted for in the analysis and did not significantly affect the results. Figure 1. Serum sample measurements were performed in duplicate. Both positive and negative controls were included in each analytical run. Calibration curves were applied and demonstrated high linearity (*R*^2^ > 0.99), thereby meeting established analytical standards. Thresholds for intra-assay and inter-assay coefficients of variation (CVs) were set at 10% and 15%, respectively.

### 2.5 Stool sampling and analysis

Stool samples were collected from participants at baseline (T0) and after 2 months (T1). The analysis of fecal SCFAs was performed using ultra-performance liquid chromatography (UPLC) coupled with a high-resolution mass spectrometry (HRMS) employing parallel reaction monitoring (PRM). Fecal samples (0.1 g) were mixed with 1,000 μl of 70% isopropanol and agitated for 5 min at 4°C using a vortex mixer. Subsequently, the mixture underwent homogenization for 10 min in an ultrasonic bath and then was centrifuged at 15,000 rpm for 15 min at 4°C. Following centrifugation, 30 μl of 45 mM 2–6 chloro-1-methylpyridinium iodide, 60 μl of 20 mM trimethylamine, and 200 μl of LC-MS grade acetonitrile were added to the supernatant. The samples were then incubated at 50°C for 5 min and further treated with 100 μl of 45 mM 2-(diethylamino) ethanol for 40 min at 50°C. Next, the samples were dried using a speedvac system at 30°C for 60 min. The dried samples were reconstituted with 300 μl of 50% acetonitrile, centrifuged at 15,000 rpm for 5 min, and transferred into chromatographic vials. Standard compounds were prepared similarly using a commercial mixture of volatile fatty acids (Volatile Free Acid Mix, TraceCERT^®^ Certified Reference Material, CRM46975 VOLATILE FREE ACID, Merck KGaA, Darmstadt, Germany). A 2 μl sample was injected into an Acquity HSS T3 column (2.1 x 50 mm, 1.7 μm particle size) at 30°C. The chromatographic elution utilized a gradient of 0.1% formic acid in water (eluent A), and 0.05% formic acid in acetonitrile (eluent B) at a flow rate of 300 μl/min. The gradient program started at 0.1% B, ramped up to 45% B over 7 min, reached 99% B at 9 min, held at 99% B until 11 min, then returned to 0.1% B by 12 min, maintaining this composition until 15 min. Ionization was performed in positive ion mode using a HESI-II ion source. Key parameters included a capillary voltage of 3.5 kV, sheath gas flow rate of 35 au, auxiliary gas flow rate of 10 au, sweep gas flow rate of 3 au, and an RF S-lens level of 50. The ion transfer tube and auxiliary gas temperatures were set to 320°C and 350°C, respectively. PRM settings included an AGC target of 5 x 10^4^ ions, a maximum injection time of 200 ms, an isolation window of 1 m/z, and a collision energy of 25% for normalized collision energy.

Zonulin concentration in stool samples were analyzed using the IDK^®^ Zonulin (Stool) Immundiagnostik kit (K 5600), with a test sensitivity of 0.118 ng/ml, following the manufacturer's protocol. Stool sample measurements were performed in duplicate, with both positive and negative controls included. Analogous to the analysis of blood samples, calibration curves with *R*^2^ > 0.99 were applied. Thresholds for intra-assay and inter-assay CVs were set at 10% and 15%, respectively.

### 2.6 Statistical analysis

The statistical analysis was performed using a PQStat software v.1.8.4 (PQStat Software, Poznan, Poland) and a GraphPad Prism 8 software (San Diego, CA, USA). Quantitative variables, which have not followed a normal distribution, were presented using median (Me) and range (min-max). Exact Fisher's test was applied to calculate the statistically significant difference in case of two binary variables in a contingency table. To determine if there were statistically significant differences between two or more Groups of an independent variable which did not meet the normal distribution criteria, the Kruskal-Wallis test was applied. The Wilcoxon test was used to compare the data collected before and after intervention. Statistical significance was determined using a threshold *p*-value < 0.05.

## 3 Results

### 3.1 Study population

A total of 47 IBS patients assigned to three Groups: Group I (*n* = 14), Group II (*n* = 14), and Group III (*n* = 19) were enrolled in the study, and encompassed 3 subtypes of IBS. These included IBS with constipation predominant (IBS-C), with diarrhea predominant (IBS-D), and IBS with mixed subtype (IBS-M). The basic characteristics of the participants before intervention were presented in Table 1. The Groups did not differ significantly in age, gender distribution or subtype of IBS.

**Table 1:** Baseline characteristics of study Groups.

**Parameter**	**Group I**	**Group II**	**Group III**	***p*-value**
Patients, *n*	14	14	19	0.5875
**Sex**, ***n***				
Women	12	10	18	0.2204
Men	2	4	1	
Age, mean (SD, min-max)	35.29 (10.96, 22–55)	37.29 (10.29, 22–56)	40.26 (9.54, 25–56)	0.2752
**Subtypes of IBS**, ***n*** **(%)**				
Mixed (IBS-M)	6 (42.9)	1 (7.1)	1 (5.3)	0.0740
Diarrhea (IBS-D)	3 (21.4)	7 (50.0)	8 (42.1)	
Constipation (IBS-C)	5 (35.7%)	6 (42.9)	10 (52.6)	

*p*-value calculated using chi-squared test (age) and Fisher's exact test (sex, IBS type), SD, standard deviation.

### 3.2 Effect of supplementation on IBS patients' quality of life

**Figure 2:**
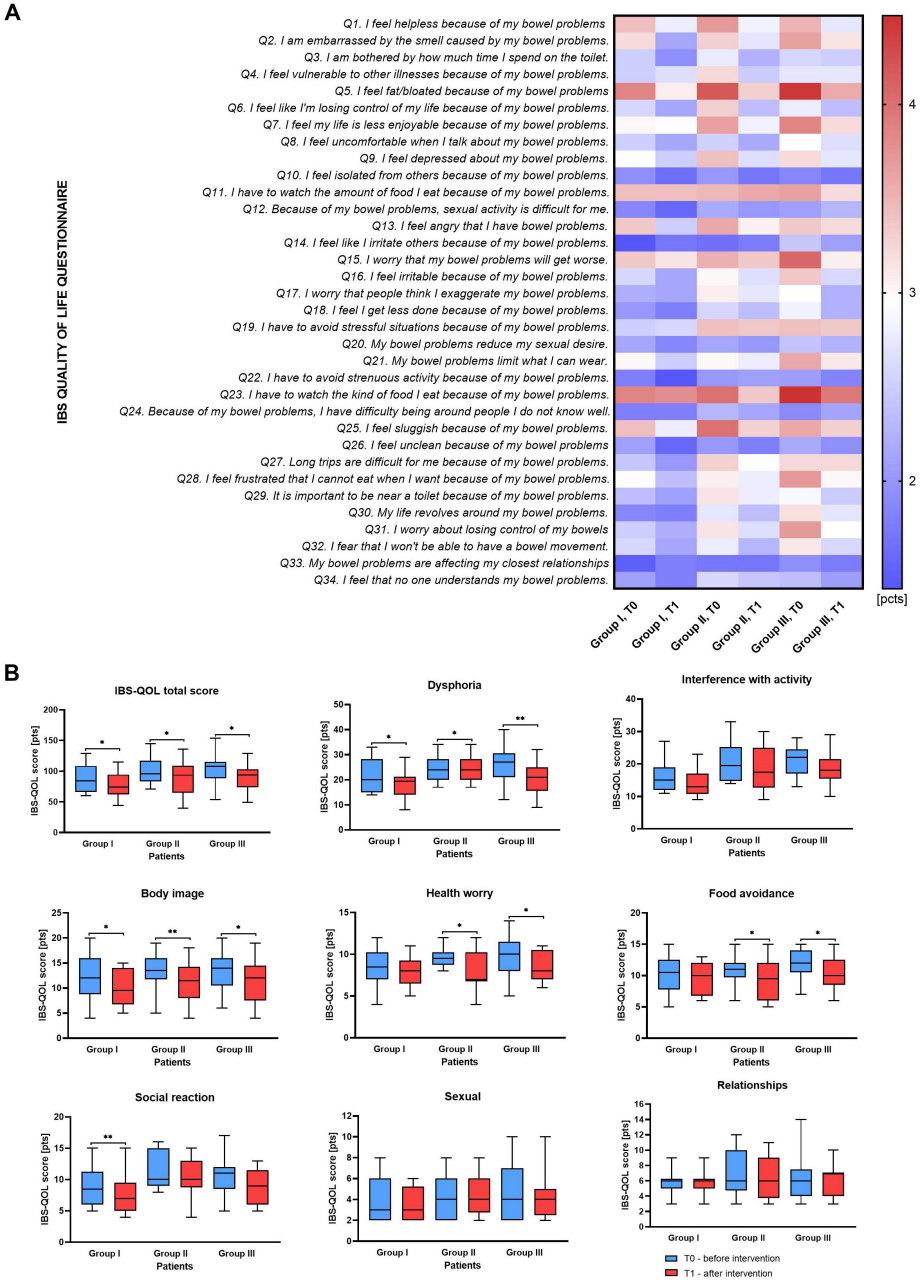
IBS-QoL score comparison results for studied patients Group before and after intervention. **(A)** heatmap with mean values of received punctation for each 34 IBS-QoL items. **(B)** plots for eight IBS-QoL score subgroups. The boxes represent the mean values with Q1–Q3 and min-max after statistical comparison performed by Wilcoxon test, *indicates statistical significance at *p* < 0.05, **p < 0.01.

The results of IBS-QoL questionnaire data presented a general downward trend in values between T0 and T1 in all studied Groups (Figure 2A), with the most pronounced improvement in total IBS-QoL survey scores observed in Group III (median of differences: −11 points, *p* = 0.0183, Supplementary Table S1). Changes in the remaining Groups, I and II were less pronounced (median of differences: −9, *p* = 0.0295 and −9.5 points, *p* = 0.01 respectively, Supplementary Table S1).

The questionnaire data, analyzed into eight subscales: (1) dysphoria, (2) interference with activity, (3) body image, (4) health worry, (5) food avoidance, (6) social reaction, (7) sexual, and (8) relationships, also demonstrated a reduction in values between T0 and T1 in all studied groups for the first six subscales (Supplementary Table S1; Figure 2B). The largest decreases in scores were observed in dysphoria in Group III (median of differences: −5, *p* = 0.0021), followed by Group II (median of differences: −3, *p* = 0.0155), and control Group I (median of differences: −1, *p* = 0.0338) (Figure 2B). Notably, all groups showed significant improvements in body image concerns. Health worry and food avoidance scores significantly decreased in groups II and III, while no changes were observed in control Group I. Social reaction scores improved only in the control Group (*p* = 0.0050). Interestingly, interference with activity, sexual function and relationship-related scores did not show significant changes across the Groups. Moreover, in Group III, significant improvements were observed in responses to 11 out of the 34 questions assessed in the IBS-QOL questionnaire. These findings suggest that adding polyphenol rich blend to probiotic and PHGG supplementation may have a beneficial impact on various aspects of life for patients with IBS.

### 3.3 Effect of supplementation on serum and stool markers

The concentrations of IL-6, IL-8, TNF-α, I-FABP-2, and GM-CSF proteins in serum were evaluated before (T0) and after (T1) the supplementation period (Supplementary Table S2).

**Figure 3:**
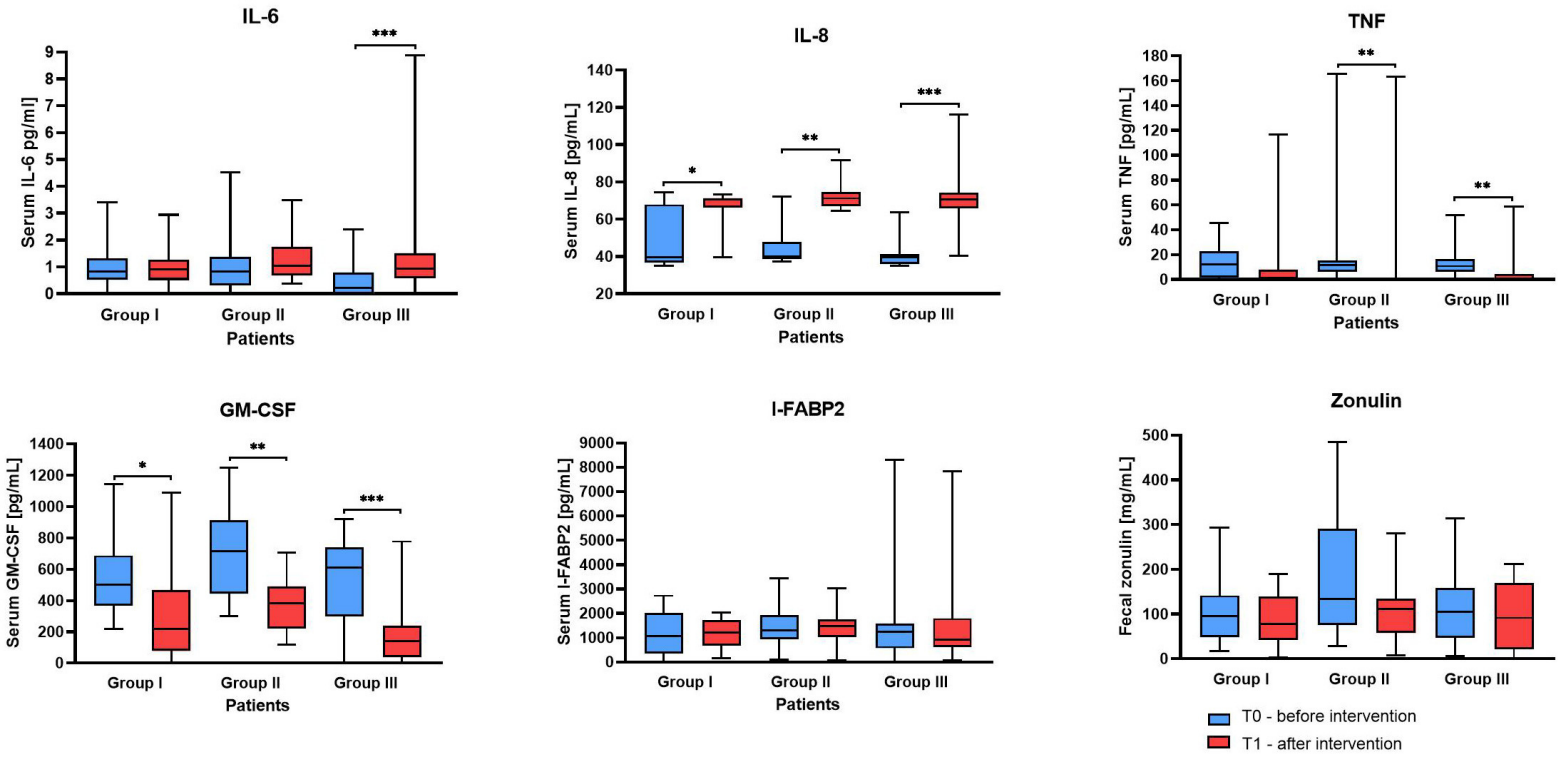
Serum markers and stool zonulin concentration before and after supplementation. The boxes represent the mean values with Q1–Q3 and min-max. Statistical differences identified by Wilcoxon test at *p < 0.05, **p < 0.01 and ***p < 0.001.

The results showed varied responses among the Groups (Figure 3). For IL-6, in Group III a significant increase from T0 to T1 (median of differences 0.59, 95% CI 0.43 to 1.11, *p*-adj. = 0.0003) was observed, whereas Groups I and II did not show significant changes. However, the IL-6 concentration in all samples, regardless of group and time point, did not exceed 5 pg/mL (except for one participant in Group III at T1). The median IL-6 levels in each group were close to 1 indicating that the IL-6 levels in the investigated participants' serum samples were within the normal range.

The IL-8 serum concentration values before the intervention in all Groups were similar and were within the range between 35 and 75 in Group I, 37 and 72 in Group II, and 35–64 in Group III. However, measurements at the second time point T1 showed a significant increase in each study Group, the lowest in Group I (median of differences: 28.3, *p* = 0.0310) and the highest in Group III (median of differences: 30.73, 0.0002).

The median for the initial serum tumor necrosis factor-α (TNF- α) concentration was 12.06 in Group I, 11.92 in Group II, and 11.23 in Group III. After supplementation, a statistically significant reduction of this cytokine was observed in groups II (*p* = 0.0025) and III (*p* = 0.006), but not in control Group I (*p* = 0.4099). Similarly, granulocyte-macrophage colony-stimulating factor (GM-CSF) levels markedly decreased in Groups II (*p* = 0.0012) and III (*p* = 0.0001), and the least in Group I (*p* = 0.0266). Serum intestinal-type fatty acid-binding protein 2 (I-FABP-2) and stool zonulin concentrations did not show significant changes across the Groups and in time interval. I-FABP2 levels remained within or near the reference range, consistent with studies suggesting limited utility of this marker in IBS without active inflammation (15). Although zonulin values were elevated in some individuals, all participants met Rome IV criteria for IBS, and organic diseases were clinically excluded. These elevations likely reflect increased intestinal permeability, particularly in IBS-D and post-infectious subtypes (16, 17). The lack of statistically significant change after the intervention may result from high interindividual variability, the multifactorial nature of IBS, or the limited specificity and sensitivity of current ELISA-based assays for fecal zonulin, underscoring the need for multi-marker approaches in gut barrier assessment (18).

**Figure 4:**
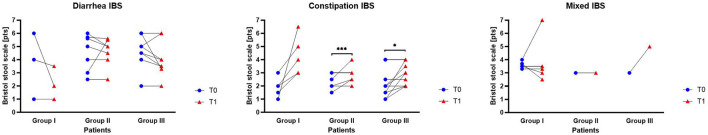
Stool consistency among IBS subtypes and studied patients Groups before and after supplementation. Circles and triangles represent individual participant results, with lines connecting each participant's pre- and post-supplementation measurements. Statistical differences identified by Wilcoxon test at *p < 0.05, and ***p < 0.001.

Stool consistency results expressed in the Bristol stool scale (BSS) among all Groups of patients before and after intervention were presented in Figure 4 and in Supplementary Table S3. In individuals with IBS-D, a decreased tendency in the BSS was observed in all groups, indicating increased stool consistency. In the case of IBS-C, the trend noted in all groups was the opposite. Specifically, after the intervention, an increase in the BSS score was observed, indicating a decrease in stool consistency (Figure 4). The highest and significant differences between T0 and T1 in IBS-C data were observed in participants from Group II (*p* = 0.0001) and Group III (*p* = 0.0312). The IBS-M form was represented by single cases in Groups II and III, therefore statistical calculations of BSS for IBS-M participants were not performed.

Analysis of the SCFAs in stool samples allowed the detection of six compounds: acetic, propionic, butyric, *n*-valeric, caproic and enanthic acid (Supplementary Table 4).

**Figure 5:**
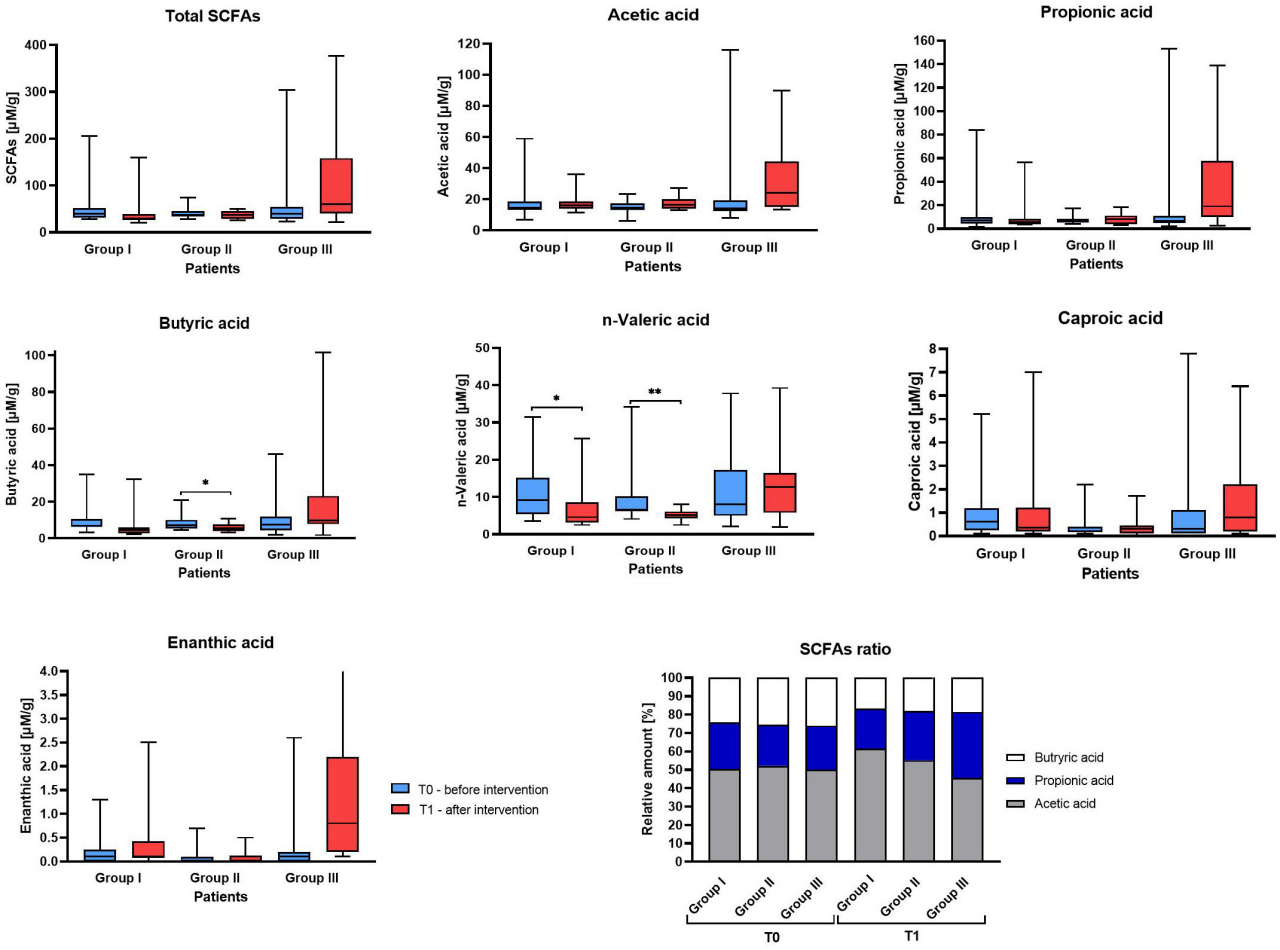
Fecal short-chain fatty acids (SCFAs) concentrations in studied IBS patient Group before and after supplementation. The boxes represent the mean values with Q1–Q3 and min-max after statistical comparison performed by Wilcoxon test, *indicates statistical significance at *p* < 0.05, **p < 0.01.

In the initial assessment at T0, no significant differences were observed in total SCFAs or in the levels of individual SCFAs across all three Groups (I, II, III) (Figure 5). Although we did not detect significant differences between T0 (baseline) and T1 (post-supplementation) within each Group, there were notable differences in SCFA concentrations at T1 between the Groups. Specifically, Group III exhibited a significantly higher concentration of total SCFAs compared to Group I at T1 (median 60.04 vs. 30.04, respectively). Group III also showed an increase in the concentrations of individual SCFAs after supplementation compared to baseline for most acids, with the highest increase observed for propionic acid (median difference: 8.09) and acetic acid (median difference: 6.89), while a slight decrease was noted for enanthic acid (median difference: −0.001). In contrast, in the control group (Group I), most SCFAs showed a decreasing trend (negative median differences), with the largest reduction observed in butyric acid (−3.27). In Group II, an increase acetic acid was observed (median difference: 1.67), while butyric and valeric acids decreased (median differences: −1.844 and −2.220 for *n*-valeric acid, respectively). The concentrations of the remaining SCFAs remained similar between the two time points. The data on the ratio of acetic acid to propionic acid to butyric acid revealed differences in relative abundances between groups in post-supplementation stool samples. Specifically, the proportion of acetic acid was lower in Group III compared to the control group and Group II (45.6% vs. 61.6% vs. 55.2%), while the proportion of propionic acid increased in Group III relative to the other groups (35.9% vs. 21.5% vs. 26.7%, respectively). In the pre-supplementation measurements, the ratio in all groups was very close to 50:25:25 (Figure 5).

## 4 Discussion

The gut-brain axis is a complex, bidirectional communication network between the intestine and central nervous system (CNS), driven by metabolic, immunological, hormonal, neural and microbial signals. In recent years, accumulating evidence has shown that the dysregulation of this axis has been associated with increased stress, anxiety and depression, markedly affecting quality of life. Moreover, physiological stress has been involved in sensitivity, permeability and composition of enteric microbiota, as well as in immune system maturation and reactivation (19, 20). Given the debilitating effect of IBS on patient's quality of life and the limited efficacy of pharmacological management, a vast majority of individuals have begun exploring additional remedies such as lifestyle changes, dietary modifications and psychological therapies. These approaches underscore the importance of comprehensive and tailored care in patients with IBS. The aim of our study was to evaluate the therapeutic potential of a novel synbiotic formulation comprising of PHGG, selected probiotic strains (*Bifidobacterium* and *Saccharomyces boulardii*), and polyphenol-rich fruit extracts blend (Fenactive^®^) in patients with IBS. To the best of our knowledge, this is the first investigation examining the effect of such formulation on both quality of life and inflammatory markers in IBS patients.

### 4.1 The effect of synbiotic formulation on quality of life

There is growing evidence, that patient reported outcomes, such as QoL and functional status play a detrimental role on the course of the disease. Individuals suffering from IBS are expected to be at high risk of impaired social interactions, poor QoL, decreased treatment adherence, impaired sexuality and mood. Notably, data has shown that patients with IBS have a three-fold increased odds of either depression or anxiety in comparison to healthy subjects (21). Furthermore, in around 50%−60% of IBS patients major psychosocial issues were reported. As such, it is of great importance to focus on the strategies that will contribute to symptom alleviation. Our study reported markedly improved QoL across various aspects of life, especially in individuals receiving supplementation (Group II and Group III). The largest decrease in dysphoria scores was noted in individuals from Group III, suggesting a positive influence of synbiotic on the emotional well-being. Furthermore, significant improvements in body image concerns were noted amongst all Groups. Health-related worries and food avoidance significantly decreased in those who received supplement, while no notable changes were observed in placebo group. Moreover, according to our analysis, the most pronounced alterations in stool consistency were seen in Group II and III. Based on the trends we can conclude that in patient with IBS-D, the reduction in BSS scores indicated more firm stools, which alleviates urgency. Conversely, in IBS-C individuals, the increased BSS scores lead to stool softening, discomfort and straining reduction, as well as ease with defecation. These findings may be explained by the unique properties of each of the used components.

One of the paramount ingredients is *B. animalis* subsp. *Lactis*, one of the most common lactic acid producing probiotics. Its superiority as the dietary supplement has been associated with significant reduction in the severity of abdominal pain, nausea, gurgling, and fluctuance (22, 23). Moreover, studies have reported increased bowel movement frequency and reduced intestinal transit time in individuals with functional constipation (24). *Bifidobacterium* spp. metabolize monosaccharides via the fructose-6-phosphate pathway to SCFAs, without gas production. This unique property may enhance tolerance for the fermentation of oligosaccharides, and consequently reduce fluctuance (25, 26). Furthermore, it has been shown to reduce abdominal discomfort and distension in individuals with IBS-C, markedly improving QoL (27).

Another important component of our supplement is fungal probiotic called *S. boulardii*, used in the treatment of various GI disorders. Clinical evidence has confirmed that *S. boulardii* reduces stool frequency, abdominal pain and distension, particularly in individuals with IBS-D (28). Studies conducted on animal models reported relief in anxiety and dysmotility after *S. boulardii* consumption. This finding may be explained by the divergent expression of the serotonin transporter (SERT)/5-hydroxytryptamine (5-HT) system in IBS subtypes, with IBS-C revealing impaired 5-HT release and IBS-D characterized by reduced 5-HT uptake and upregulated SERT activity, resulting in improved intestinal mobility (29, 30). These findings are in line with our findings, where individuals receiving supplementation reported marked reduction in anxiety and gut dysmotility. Symptom alleviation contributes to improvement in QoL by fostering emotional stability, and restoring the ability to engage in daily, social and professional activities.

PHGG is a non-gelling, water soluble fiber, that has emerged as a promising compound in relieving symptoms in diarrhea-predominant and constipation-predominant IBS. Its administration has been associated with marked improvement in abdominal tension, spasms, and fluctuance (11). Robust evidence indicates that fiber administration alleviates intensity and frequency of episodes in individuals with diarrhea. Moreover, its effectiveness in stool softening, increases bulking capacities and fecal excretory feeling, alleviates discomfort associated with defecation in persons with constipation. A multicenter randomized open trial studied the effect of PHGG on QoL in individuals with IBS (31). Improvement in the Hospital Anxiety and Depression Scale as well as in the Gastrointestinal Symptom Rating Scale (majority of the Short Form 36 items) was reported with PHGG administration. These outcomes were sustained even after 6 months of follow up. Even though it is not explicitly classified as a FODMAP, PHGG- especially the low molecular-weight form, has been shown to exert prebiotic properties similar to fructo-oligosaccharides (32, 33). Data has shown that intake of only 6 g/day of PHGG increases the concentration of beneficial intestinal *Bifidobacteria, Lactobacilli, Parabacteroidetes* and SCFAs (34–36). Studies have reported that the selective increase in these bacteria modulates intestinal microbiota, resulting in relief in abdominal bloating and pain (37). SCFAs are crucial for maintenance of enteric homeostasis and reduction of inflammation, by providing adequate colonic lining, stimulating antimicrobial peptides and mucus production (38). Furthermore, PHGG has been found to enhance insulin response and markedly reduce postprandial plasma glucose. Moreover, the ingestion of PHGG reduces serum cholesterol and triglyceride levels, boosts absorption of minerals by improving lipid metabolism without attenuation of protein utilization (39).

*Aronia melanocarpa* has the highest polyphenols concentration compared to the other plant sources. It has been found to exert numerous beneficial effects including antioxidant, antineoplastic, antidiabetic, neuroprotective and anti-infective (40). Research reported that *A. melanocarpa* extract reversed the dextran sulfate sodium (DSS)-induced intestinal dysbiosis, fostering the production of SCFAs and restoring gut microbiota homeostasis (41). Another study examined the effect of *A. melanocarpa* in a rat with trinitrobenzensulfonic acid (TNBS) induced colitis (42). Significant improvement in the microscopic and macroscopic manifestations of colitis was noted, underlying its potent anti-inflammatory and antioxidant properties. Interestingly, study reported that the effect of *A. malenocapra* fruit juice is compared to or even higher than that of sulfasalazine.

*Sambucus nigra* has been found to play an important role in the regulation of immune system. It downregulates the expression of pro-inflammatory genes, reduces increased production of inflammatory mediators, consequently inhibiting pro-inflammatory pathway in LPS-stimulated macrophages (43). Interestingly, its administration increased mucosal layer thickness, number of mast cells and goblet cells in colon tissue and reduced the expression of TNF-α (44). Moreover, dietary incorporation prevented astrocyte reactivity and astrogliosis. These findings support the evidence that *S. nigra* is a potent antioxidant and immune-modulatory compound, providing support to gut-brain axis. These unique properties ameliorate classic IBS symptoms such as cramping, abdominal pain, bloating.

Our findings closely align with those reported in other studies investigating the effect of low-FODMAP diet on QoL in patients with IBS. In a study by Guerreiro et al. (45) a low-FODMAP diet was associated with markedly reduced negative effects of IBS on body image, sexual life, dysphoria, interference with daily activities and interpersonal connections. Moreover, significant relief in diarrhea and abdominal pain were reported. Similar results were observed in a clinical trial conducted by Naseri et al. (46) where dietary intervention was associated with 30%−60% reduction in IBS symptom severity. Recent systematic review and meta-analysis comprising of 76 RTCs examining the efficacy of probiotics and low FODMAP diet in IBS patients, revealed alleviation of global IBS symptoms and reduction of abdominal pain score (47). Another study noted that in individuals following a low-FODMAP diet, abdominal pain decreased by 60%, fluctuance by 87.5%, and bloating by 70% (48). Additionally, improvement in stool formation in IBS-D individuals was reported. In the study performed by Ankersen et al. (49) the low-FODMAP diet has been shown to alleviate GI symptoms and improve bowel habits by reducing stool frequency and enhancing consistency. A further investigation comparing low-FODMAP diet with a high-FODMAP diet revealed statistically significant improvement in burping reduction, exhaustion, and regurgitation in low-FODMAP intervention (50). Data presented by Bohn et al., demonstrated that low-FODMAP diet led to significant improvement in the overall IBS symptoms, including reduced abdominal pain, and fewer bowel movements by day 29 of therapy, whereas no changes were noted in the traditional IBS diet (51). Similar findings were reported by Conley et al. (52), where following low-FODMAP restriction diet, the magnitude of pain and overall symptom alleviation were greater in those with distinct IBS microbiome, compared to healthy ISB controls (52). The aforementioned studies provide robust evidence that low-FODMAP diet not only improves the QoL of patients with IBS compared with those following standard dietary recommendations or a high-FODMAP diet, but also has favorable effects on relieving abdominal pain, diarrhea, bloating and stool frequency.

### 4.2 The effect of synbiotic formulation on inflammatory markers

It is well known that low grade intestinal inflammation plays an important role in the pathophysiology of IBS leading to baseline elevation of inflammatory cytokines. The signaling of IL-6, a versatile cytokine is of particular interest, as it features a complex network of numerous pathways and modes of activation in targeted cells. Several mouse models have shown that regenerative and anti-inflammatory properties are mediated by classic signaling, whereas pro-inflammatory are interceded by trans signaling (53). Considering this intricacy, IL-6 plays a two-fold role in the intestine, and deviations of IL-6 signaling and concentrations may affect the homeostasis. Study reported that, in a murine colon cancer model leads to restoration of intestinal epithelial cells, and inhibition of epithelial cell apoptosis, thus enhancing barrier function (54). Notably, under physiological conditions, enteric microbiota stimulates the intraepithelial lymphocytes to release IL-6, required for maintenance of equilibrated intestinal epithelial permeability and mucin production (55). Furthermore, IL-6 is essential for perpetuation of the stem cell microenvironment and epithelial cell proliferation required for wound healing, following intestinal injury (56). Our data demonstrates significantly increased levels of IL-6 in Group III after synbiotic administration, though this value remain within the normal range. This combination may indicate its beneficial effect on modulating immune response, facilitating tissue repair and enhancing intestinal barrier integrity.

IL-8 is another important multifunctional chemokine, widely recognized for its neutrophil chemoattractant properties, engaged in acute inflammation. More recently, its actions are also being acknowledged in tissue remodeling, angiogenesis, and regulation of epithelialization (57, 58). Comparable results were reported by Maheshwari et al. (59), who examined the effect of IL-8 on the developing human intestine. Study suggested that apart from the neutrophil chemotaxis properties, IL-8 exerts a trophic function in the developing human intestine. In our study the greatest rise in IL-8 levels was noted in Groups II and III. This finding may advocate for a transient and favorable immune activation aimed at restoring gut homeostasis, underscoring the imperative role of IL-8 as a potent immunomodulatory chemokine. Although post-treatment IL-8 levels slightly exceeded the general reference value of 66.1 pg/mL, this elevation appears to reflect a localized and transient immune activation rather than systemic inflammation. Such controlled IL-8 upregulation has been associated with epithelial regeneration and mucosal healing processes, supporting its potential role in restoring gut homeostasis (60, 61).

TNF-α is an inflammatory cytokine produced by macrophages/monocytes during acute inflammatory states. It may commence apoptosis, regulate cell survival and proliferation. Studies demonstrated higher levels of TNF-α in IBS patients compared to healthy controls. A positive correlation between plasma levels of TNF-α and fatigue in patients with IBS has been reported (62). Our results indicated significant reduction of TNF-α in both patients who received probiotic strains (*Bifidobacterium and Saccharomyces boulardii*) and PHGG (Group II) and those who were administered probiotic strains, PHGG and Fenactive^®^ blend. Analogous findings were identified in RCT, which examined the effect of *S. boulardii* in individuals with IBS-D. Significant decrease in TNF-α was observed with probiotic administration compared to placebo (63). In another study, treatment with *B. animalis* ssp. *Lactis* 420 markedly reduced the expression of TNF-α (64). In a subsequent investigation, PHGG intake was associated with a significant suppression of lipopolysaccharide-induced TNF-α production, which markedly reduced intestinal inflammation (65).

GM-CSF is a fundamental regulator of intestinal macrophage activation in individuals with IBS. It has been found to promote polarization and maturation of inflammatory enteric macrophages, facilitating anti-microbial properties while suppressing wound healing (66). Our study revealed markedly reduced levels of GM-CSF in Groups II and III. This finding may suggest improvement in intestinal barrier integrity and reduction in inflammatory processes. This is in line with another study which reported that *S. boulardii* inhibits the mRNA expression related to GM-CSF and TNF-α (67). Another study examined the effect of *Saccharomyces cerevisiae boulardii* on intestinal epithelial and dendritic cells *in vitro* in the context of enterotoxigenic *Escherichia coli* (ETEC) infection (68). Presented data revealed reduced mRNA ETEC-induced gene expression of pro-inflammatory cytokines such as GM-CSF and TNF-α. Moreover, in porcine intestine, *S. boulardii* has been found to reduce the adhesion of ETEC to host intestinal cells, decrease bacterial internalization and enhance pathogen elimination. In accordance to current data, probiotic *Saccharomyces* has been shown to act as an immunomodulator, exerting anti-inflammatory properties, alleviating abdominal pain, and improving QOL (69).

There is a limited body of research focusing on the effect of low-FODMAP diet on inflammatory markers, particularly interleukins, GM-CSF or TNF-α. Tuck et al. examined the effects of low- and high-FODMAP in a murine model of DDS-induced colitis across three groups (70). While a high-FODMAP diet was associated with increased inflammatory markers such as myeloperoxidase activity (an indicator of neutrophil infiltration), the data suggests that variations in dietary FODMAP intake did not exacerbate or mitigate overt inflammation. No significant differences were observed in key cytokines, including TNF-α, IL-1β, IL-10, and GM-CSF, despite triggering shift toward proteolytic fermentation in the post-inflammatory state (70). On a contrary, in a different study comprising of 20 IBS patients, serum levels of IL-6 and IL-8 decreased significantly (50). The impact on inflammatory markers remains underexplored and warrants further investigation.

### 4.3 The effect of synbiotic formulation on SCFAs

SCFAs are produced by commensal bacteria through fermentation of partially and non-digestible polysaccharides, of which acetate, propionate and butyrate are most abundant (>95%) (71, 72). They are most prevalent in the proximal colon, where they are utilized by enterocytes to induce reactive oxygen species, alter chemotaxis and phagocytosis. Enteric SCFAs production is pivotal for maintenance of gut homeostasis, by mucus production, regulation of the luminal acid-base balance, and immunomodulation (73). Numerous clinical studies have confirmed, that SCFAs exert antimicrobial, anti-inflammatory, antineoplastic effect. SCFAs, especially butyrate have been reported to alter the secretion of pro-inflammatory cytokines including IL-6, IL-8, TNF-α. Moreover, SCFAs are involved in the differentiation of regulatory T cells and effector T-cells. Propionate and butyrate have been involved in the enhancement of intestinal barrier integrity (74). Another study reported that SCFAs have significant effect on colonic motility. In the proximal colon, butyrate was found to increase the frequency of contractions, whereas acetate and propionate decreased the frequency of contractions. Concurrently, in the distal colon, the rate of colonic propulsion was increased by butyrate, and decreased by propionate (75). Our analysis revealed significantly higher concentrations of total SCFAs in Group III compared to Group I after intervention, with greatest increase noted in propionic acid and acetic acid. In Group II, an increase in acetic acid was observed, while butyric and valeric acid decreased. These findings underline that this novel synbiotic formulation has beneficial effect on intestinal barrier integrity, motility and immunity. In comparison, among 20 patients with IBS-D or mixed IBS who received low-FODMAP intervention, total SCFAs and n-butyric acid decreased compared to baseline, however no other significant changes in SCFA levels were noted across baseline after low-FODMAP diet or high-fructo-oligosaccharide supplementation (50). According to other research articles, no difference between total SCFA or individual SCFAs was noted after low-FODMAP intervention (70, 76). Whilst Conley et al. reported significant reduction in SCFAs after FODMAP restriction in IBS individuals with dysbiotic microbiome compared to microbiome resembling healthy controls (52). In authors work, a metabolite-based model identified microbial subtypes, supporting targeted FODMAP restriction by balancing the efficacy with treatment burden. It was suggested that SCFA recalibration rather than depletion, while attenuated shifts post-reintroduction phase might reflect lasting microbiome changes or selective avoidance of fermentable foods, warranting future studies.

It is acknowledged that this study has certain limitations. Firstly, the relatively small size of patients enrolled in our analysis. Secondly, the short duration of the intervention period, restricting the ability to assess the long-term effects of synbiotic supplementation. Thus, further research with a larger cohort is warranted to validate our results and elucidate further impact of synbiotic. Moreover, given the substantial effect of gut microbiota in the pathogenesis of IBS, profiling of intestinal microbiome would be beneficial to incorporate in future research. This would allow more comprehensive overview of the underlying mechanisms underpinning the outcomes of the intervention. One notable limitation of this study is the selection of regular maltodextrin as the placebo. While it is frequently used in clinical research, accumulating evidence suggests that maltodextrin may exert biological effects rather than acting as a truly inert substance. A systematic review showed that around 60% of randomized controlled trials utilizing maltodextrin reported measurable impacts on physiological parameters or gut microbiota, including alterations in immune function and gastrointestinal physiology (77). These findings raise important questions regarding its appropriateness as a placebo in studies involving gastrointestinal conditions such as IBS. Although the maltodextrin used in our study was verified as non-resistant and not considered a functional ingredient (CoA included in the supplementary material), the possibility of subtle gut-related effects cannot be entirely dismissed. Future investigations should consider alternative placebo agents with well-established inertness in the context of gut health research.

## 5 Conclusions

Our results indicate that supplementation with a combination of *B. animalis* subsp. *lactis* BLC1, *S. boulardii* SP9, *B. lactis* UABla-12TM, *B. animalis* subsp. *lactis* BS01, PHGG, and Fenactive^®^ significantly improves QoL in patients with IBS. The largest decrease in dysphoria scores was noted in individuals from Group III, suggesting a positive influence of synbiotic on the emotional well-being. Health-related worries and food avoidance significantly decreased in those who received intervention, while no notable changes were observed in placebo group. In patients with IBS-D, the reduction in BSS scores indicated more firm stools, which alleviates urgency. Conversely, in IBS-C individuals, the increased BSS scores lead to stool softening, discomfort and straining reduction, as well as ease with defecation. All participants were rigorously diagnosed with IBS according to Rome IV criteria, with organic, autoimmune, and inflammatory diseases being excluded. Although some patients showed elevated baseline levels of pro-inflammatory cytokines such as IL-8, TNF-α, and GM-CSF—consistent with low-grade immune activation in IBS-D and post-infectious IBS; the observed post-intervention immune response likely reflects a regulatory effect (78, 79). The greatest rise in IL-8 levels was noted in Groups II and III, advocating for a transient and favorable immune system activation aimed at restoring gut homeostasis. Concurrent reductions in TNF-α and GM-CSF further support the anti-inflammatory and immunomodulatory potential of the synbiotic formulation. Additionally, higher concentrations of SCFAs were detected in patients receiving the synbiotic compared to placebo. Based on these findings, supplementation with polyphenols, selected probiotic strains, and PHGG appears to be a promising approach for alleviating IBS symptoms and supporting gut health.

## Data Availability

The original contributions presented in the study are included in the article/Supplementary material, further inquiries can be directed to the corresponding authors.
