# Supplementary material for: Effects of a polyphenol-rich extract blend, probiotics, and hydrolyzed fiber on quality of life and gut health markers in patients with irritable bowel syndrome—A randomized, double-blind, placebo-controlled trial
Source: Front Nutr. 2025 Jul 7;12:1603011. doi: 10.3389/fnut.2025.1603011 (PMC12277158; doi:10.3389/fnut.2025.1603011)
Supplement: Supplementary file 1 [file Data_Sheet_1.pdf]

Supplementary Material

Supplementary tables

Supplementary Table S1. IBS-QoL scores in studied IBS patients' groups before and after supplementation.

		GROUP I, n = 14					GROUP II, , n = 14					GROUP III, , n = 19				
IBS-QOL		T0	T1	Me_ diff.	95% CI	p -value	T0	T1	Me_ diff.	95% CI	p -value	T0	T1	Me_ diff.	95% CI	p -value
Overall		87.4 (23.4)	76.8 (20.2)	-9.00	1.24; 20.04	0.0295	101.0 (20.2)	89.6 (28.4)	-9.50	3.23; 19.63	0.0100	103.3 (22.6)	88.7 (22.7)	-11.00	-25.5; 0.00	0.0183
SUBSCALES	Dysphoria	20.0 (14.0-33.0)	19.5 (8.0-29.0)	-1.00	-7.00; -1.00	0.0338	24.0 (17.0-34.0)	24.0 (17.0-34.0)	-3.00	-6.50; -1.00	0.0155	27.0 (12.0-40.0)	21.0 (9.0-32.0)	-5.00	-8.5; -2.0	0.0021
	Interference with activity	15.0 (11.0-27.0)	13.0 (9.0-23.0)	-1.50	-5.50; 0.50	0.1223	19.5 (14.0-33.0)	17.5 (9.0-30.0)	-1.00	-4.50; 1.00	0.2537	22.0 (13.0-28.0)	18.0 (10.0-29.0)	-2.00	-5.00; -0.50	0.0867
	Body image	12.0 (4.0-20.0)	9.5 (5.0-15.0)	-2.50	-4.50; -1.00	0.0154	13.5 (6.0-20.0)	11.5 (4.0-19.0)	-2.00	-3.50; -1.00	0.0087	14.0 (6.0-20.0)	12.0 (4.0-19.0)	-2.00	-4.00; -0.50	0.0220
	Health worry	8.5 (4.0-12.0)	8.0 (5.0-11.0)	-1.00	-2.00; 1.00	0.3621	9.5 (8.0-12.0)	7.0 (4.0 -12.0)	-2.00	-3.00; 0.00	0.0285	10.0 (5.0-14.0)	8.0 (6.0-11.0)	-2.00	-2.50; -0.50	0.0102
	Food avoidance	10.5 (5.0-15.0)	10.0 (6.0-13.0)	-1.00	-0.71; 1.99	0.3228	11.0 (6.0-15.0)	9.5 (5.0-15.0)	-1.00	-3.00; 0.00	0.0285	12.0 (7.0-15.0)	10.0 (6.0-15.0)	-1.00	-3.00; 0.00	0.0449

	**Social reaction**	8.5 (5.0-15.0)	7.0 (4.0-15.0)	-1.00	-2.00; -1.00	0.0050	10.0 (8.0-16.0)	10.0 (4.0-15.0)	-1.00	-4.00; 0.00	0.0718	11.0 (5.0-17.0)	9.0 (5.0-13.0)	0.00	-4.50; 1.00	0.2887
	**Sexual**	3.0 (2.0-8.0)	3.0 (2.0-6.0)	-0.50	-2.00; 0.50	0.1593	4.0 (2.0-8.0)	4.0 (2.0-8.0)	0.00	-2.00; 2.00	0.8902	4.0 (2.0-10.0)	4.0 (2.0-10.0)	0.00	-2.00; 1.00	0.8345
	**Relationships**	6.0 (3.0-9.0)	5.0 (3.0-9.0)	0.00	-1.50; 1.00	0.8066	6.0 (3.0-12.0)	6.0 (3.0-11.0)	-0.50	-2.00; 1.00	0.3358	6.0 (3.0-14.0)	7.0 (3.0-10.0)	0.00	-4.50; 1.00	0.8359

Data expressed as median (Me) and minimum and maximum values (min-max), and median of differences (Me_diff.) with 95% confidence interval (95%CI). The statistical results (p-value) calculated with the non-parametric Wilcoxon test to compare T0 and T1 dependent groups or t-student test. An exception is overall punctation were data presented are as mean with standard deviation and calculation was performed using t-student test. Red font - statistically significant results ($p < 0.05$).

IBS-QoL Domains and Corresponding Questionnaire Items

Domain	Questionnaire Items (Q#)
Dysphoria	Q1, Q6, Q7, Q9, Q10, Q13, Q16, Q30
Activity Interference	Q3, Q18, Q19, Q22, Q27, Q29, Q31
Body Image	Q5, Q21, Q25, Q26
Health Worry	Q4, Q15, Q32
Food Avoidance	Q11, Q23, Q28
Social Reaction	Q2, Q14, Q17, Q34
Sexual Concerns	Q12, Q20
Relationships	Q8, Q24, Q33

Supplementary Table S2. Effect of supplementation on serum markers and stool zonulin in studied IBS patients' groups.

	PARAMETER	GROUP	T0			T1			T0 vs T1		
			Me	Min-max	p-value	Me	Min-max	p-value	Me_ diff.	95%CI	p-value
SERUM	**IL-6 [pg/mL]**	I	0.84	0.0-3.41	0.0472	0.91	0.0-2.94	0.5918	0.07	-0.62; -0.54	0.7798
		II	0.82	0.0-4.52		1.05	0.37-3.49		0.27	-0.58; 1.07	0.5300
		III	0.21	0.0-2.41		0.93	0.0-8.88		0.59	0.43; 1.07	0.0003
	**IL-8 [pg/mL]**	I	39.71	35.13-74.55	0.1996	70.68	39.5-73.26	0.5	28.3	-3.87; 34.16	0.0310
		II	39.97	37.42-72.29		71.17	64.55-91.65		29.53	5.49; 37.84	0.0017
		III	39.6	35.02-63.58		70.68	40.44-116.16		30.73	27.62; 35.36	0.0002
	**TNF-α [pg/mL]**	I	12.06	0.0-45.61	0.9549	1.26	0.0-116.5	0.1647	-11.5	-20.32; 12.33	0.4099
		II	11.92	0.0-165.4		0	0.0-163.0		-9.28	-15.11; -6.94	0.0025
		III	11.23	0.0-51.78		0	0.0-58.65		-11.22	-15.67; -7.75	0.0060
	**GM-CSF [pg/mL]**	I	500.78	219.14-1142.24	0.2581	218.68	0.0-1089.29	0.0099	-266.76	-412.73; -21.42	0.0266
		II	714.88	299.14-1249		382.76	117.77-704.59		-335.09	-532.66; -157.94	0.0012
		III	610.47	0.0-919.59		138.68	0.0-776.35		-469.81	-535.87; -233.80	0.0001
	**I-FABP-2 [pg/mL]**	I	1076.43	0.0-2725	0.8143	1217.86	160.71-2032.14	0.5238	160.71	-444.29; 340.36	0.9460
		II	1312.15	87.14-3442.86		1478.57	67.86-3028.57		77.14	-726.4; 502.1	0.9001

		**III**	1251.43	0.0-8301.43		914.29	64.29-7846.43		-153.21	-401.43; 186.07	0.4171
**STOOL**	**ZONULIN [ng/mL]**	**I**	94.83	16.6-293.5	0.3179	78.43	3.325-190.15	0.8556	-16.88	-73.96; 40.30	0.5016
		**II**	133.05	28.49-484.5		111.25	6.835-280.15		-76.10	-150.4; 0.20	0.0580
		**III**	104.45	5.4-313.85		91.2	0.0-211.15		-18.92	-58.89; 18.83	0.3525

Data expressed as median (Me) and minimum and maximum values (min-max), and median of differences (Me_diff.) with 95% confidence interval (95%CI). The statistical results (p-value) calculated with the non-parametric Kruskal-Wallis test to compare three groups comparison and Wilcoxon test to compare T0 and T1 dependent groups. Red font - statistically significant results ($p < 0.05$).

Supplementary Table S3. Stool consistency among IBS subtypes before and after supplementation.

**IBS-subtype**	**GROUP**		**T0**		**T1**		**T0 vs T1**		
			**Me**	**Min-max**	**Me**	**Min-max**	**Me_diff.**	**95%CI**	**p-value**
**Diarrhea, n=18**	**I**	n = 3	2.00	1.00-6.00	4.00	1.00-3.50	-0.50	-2.25, 2.25	0.3700
	**II**	n = 7	5.00	2.50-6.00	5.00	2.50-5.50	-0.10	-0.75, 1.00	0.5900
	**III**	n = 8	4.75	2.00-6.00	3.75	2.00-6.00	-0.5	-1.85, 0.25	0.2020
**Constipation, n = 21**	**I**	n = 5	2.00	1.00-3.00	4.00	3.00-6.50	2.00	1.00-5.50	0.0625
	**II**	n = 6	2.00	1.50-3.00	3.00	2.00-4.00	0.25	0.50-1.50	0.0001
	**III**	n = 10	2.00	1.00-4.00	3.00	2.00-4.00	0.75	0.75-2.00	0.0312
**Mixed, n = 8**	**I**	n = 6	3.50	3.30-4.00	3.40	2.50-7.00	3.0	-1.73,1.30	0.7280
	**II**	n = 1	3.00	3.00	3.00	3.00	NA	NA	NA
	**III**	n = 1	3.00	3.00	5.00	5.00	NA	NA	NA

Data expressed as median (Me) and minimum and maximum values (min-max), and median of differences (Me_diff.) with 95% confidence interval (95%CI). The statistical result (p-value) calculated with Wilcoxon test to compare T0 and T1 dependent groups. Red font - statistically significant results ($p < 0.05$).

Supplementary Table S4. Fecal short-chain fatty acids (SCFAs) concentrations in studied IBS patient group before and after supplementation.

	GROUP	T0			T1			T0 vs T1		
		Me	Min-max	p-value	Me	Min-max	p-value	Me_diff.	95%CI	p-value
Total SCFA [µM/g]	I	40.10	27.95-205.00	0.9540	30.04	20.48-160.2	0.0050	-3.88	-27.33; 2.32	0.1350
	II	37.00	28.39-74.23		36.50	25.43-49.36		0.00	-23.16; 9.24	0.5420
	III	39.90	23.05-303.5		60.04	21.46-376.1		1.29	-5.83; 85.98	0.4560
Acetic acid [µM/g]	I	14.48	6.84-58.91	0.7474	16.06	11.64-36.16	0.1323	-0.06	-4.38; 10.92	0.9515
	II	14.79	5.93-23.21		16.55	13.03-27.08		1.67	-1.66; 8.96	0.2958
	III	14.29	8.05-115.8		24.24	13.47-89.89		6.89	-3.64; 16.34	0.1688
Propionic acid [µM/g]	I	7.26	1.49-83.98	0.5827	5.59	3.28-56.44	0.0040	1.42	-5.94; 5.06	0.4263
	II	6.25	4.09-17.44		7.99	3.09-18.50		-0.01	-1.97; 5.76	0.5416
	III	6.79	2.07-153.0		19.09	2.50-139.00		8.09	-1.78; 34.57	0.1336
Butyric acid [µM/g]	I	6.92	3.40-34.99	0.9229	4.41	2.43-32.27	0.0046	-3.27	-8.79; 1.42	0.1040
	II	7.22	4.72-20.88		5.45	3.26-10.65		-1.84	-4.02; 1.31	0.0353
	III	7.41	2.03-46.10		9.82	1.83-101.50		1.09	-5.31; 12.74	0.3525
n-Valeric acid [µM/g]	I	9.20	3.61-31.43	0.8180	4.56	2.45-25.75	0.0335	-2.72	-13.19; -0.10	0.0245
	II	6.64	4.05-34.06		5.14	2.51-7.98		-2.22	-6.18; 0.11	0.0085
	III	8.00	2.06-37.78		12.66	1.88-39.18		0.25	-9.11; 9.11	0.8288
	I	0.64	0.11-5.24	0.1838	0.38	0.08-7.03	0.0511	-0.00	-1.40; 0.66	0.9515

**Caproic acid [µM/g]**	**II**	0.21	0.08-2.02		0.28	0.00-1.66		-0.03	-0.36; 0.16	0.6257
	**III**	0.28	0.12-7.81		0.84	0.06-6.40		0.25	-0.09; 1.50	0.1346
**Enanthic acid [µM/g]**	**I**	0.12	0.0-1.28	0.3151	0.11	0.02-2.51	0.0901	0.03	-0.15; 0.37	0.7869
	**II**	0.04	0.02-0.73		0.04	0.01-0.48		-0.01	-0.04; 0.03	0.4263
	**III**	0.05	0.02-2.61		0.05	0.014-2.64		-0.00	-0.02; 0.43	0.5412

Data expressed as median (Me) and minimum and maximum values (min-max), and median of differences (Me_diff.) with 95% confidence interval (95%CI). Statistical result (p-value) calculated with the non-parametric Kruskal-Wallis test for three groups comparison and Wilcoxon test for T0 and T1 dependent groups comparison. Red font - statistically significant results ($p < 0.05$).
